# Clinical performance of the next generation Elecsys Troponin T high-sensitivity Gen 6 assay in acute coronary syndrome (PERFORM-TSIX): study design

**DOI:** 10.1007/s00392-025-02842-x

**Published:** 2026-01-19

**Authors:** Lori B. Daniels, Evangelos Giannitsis, Christian Mueller, Steven J. R. Meex, David Buehlmann, Dunja Kurtoic, Garnet Bendig, Mette Cole, Richard Body, Robert H. Christenson, Christa Cobbaert, Christopher R. deFilippi, Kai M. Eggers, Kenji Inoue, Allan S. Jaffe, Cian P. McCarthy, James McCord, Johannes T. Neumann, Torbjørn Omland, Cynthia Papendick, Yader Sandoval, Jack Wei Chieh Tan, Martin P. Than, Raphael Twerenbold, Nicholas L. Mills, W. Frank Peacock

**Affiliations:** 1 Division of Cardiovascular Medicine, Department of Medicine, University of California, San Diego, La Jolla, CA USA; 2 Department of Internal Medicine III, Cardiology University Hospital of Heidelberg, Heidelberg, Germany; 3 Cardiovascular Research Institute Basel (CRIB) and Department of Cardiology, University Hospital Basel, University of Basel, Basel, Switzerland; 4 Central Diagnostic Laboratory, Maastricht University Medical Center, Maastricht, the Netherlands; 5 CARIM School for Cardiovascular Diseases, Maastricht University, Maastricht, the Netherlands; 6 Clinical Development, Roche Diagnostics International AG, Rotkreuz, Switzerland; 7 Biostatistics, Roche Diagnostics International AG, Rotkreuz, Switzerland; 8 Clinical Operations, Roche Diagnostics GmbH, Penzberg, Germany; 9 Clinical Operations, Roche Molecular Systems, Inc., Indianapolis, IN USA; 10 Emergency Department, Manchester University NHS Foundation Trust, Manchester Academic Health Science Centre, Manchester, UK; 11 Division of Cardiovascular Sciences, University of Manchester, Manchester, UK; 12 Department of Pathology, University of Maryland School of Medicine, Baltimore, MD USA; 13 Department of Clinical Chemistry and Laboratory Medicine, Leiden University Medical Center, Leiden, the Netherlands; 14 Departments of Medicine and Pathology, University of Maryland School of Medicine, Baltimore, MD USA; 15 Department of Medical Sciences, Uppsala University, Uppsala, Sweden; 16 Tokyo Heart Rhythm Clinic Shinjuku, Tokyo, Japan; 17 Department of Cardiovascular Biology and Medicine, Juntendo University Graduate School of Medicine, Tokyo, Japan; 18 Department of Cardiovascular Medicine and Department of Laboratory Medicine and Pathology, Mayo Clinic, Rochester, MN USA; 19 Heart and Vascular Institute, Massachusetts General Brigham and Harvard Medical School, Boston, MA USA; 20 Heart and Vascular Institute, Henry Ford Health, Detroit, MI USA; 21 Department of Cardiology, University Heart and Vascular Centre Hamburg, University Medical Center Hamburg-Eppendorf, Hamburg, Germany; 22 Center for Population Health Innovation, University Heart and Vascular Center Hamburg, University Medical Center Hamburg-Eppendorf, Hamburg, Germany; 23 German Center for Cardiovascular Research, Partner Site Hamburg/Kiel/Lübeck, Hamburg, Germany; 24 Department of Epidemiology and Preventive Medicine, School of Public Health and Preventive Medicine, Monash University, Melbourne, Australia; 25 Department of Cardiology, Akershus University Hospital, Lørenskog, Norway; 26 Institute of Clinical Medicine, K. G. Jebsen Centre for Cardiac Biomarkers, University of Oslo, Oslo, Norway; 27 Department of Emergency Medicine, The Royal Adelaide Hospital, Central Adelaide Local Health Network, Adelaide, South Australia Australia; 28 Adelaide Medical School, The University of Adelaide, Adelaide, South Australia Australia; 29 Minneapolis Heart Institute, Abbott Northwestern Hospital and Center for Coronary Artery Disease, Minneapolis Heart Institute Foundation, Minneapolis, MN USA; 30 National Heart Centre Singapore, 5 Hospital Dr, Singapore, 169609 Singapore; 31 Department of Cardiology, Duke-NUS Medical School, Singapore, Singapore; 32 Department of Cardiology, Sengkang General Hospital, Singapore, Singapore; 33 Department of Emergency Medicine, Christchurch Hospital, Christchurch, New Zealand; 34 Department of Medicine, University of Otago, Christchurch, New Zealand; 35 Department of Emergency Medicine, University of Kansas Medical Center, The University of Kansas Health System, Kansas City, KS USA; 36 British Heart Foundation Centre for Cardiovascular Science, University of Edinburgh, Edinburgh, UK; 37 Department of Emergency Medicine, Baylor College of Medicine, Houston, TX USA

**Keywords:** Acute myocardial infarction, Acute coronary syndrome, High-sensitivity cardiac troponin T, Protocol, Risk stratification, Diagnosis

## Abstract

**Background:**

High-sensitivity cardiac troponin (hs-cTn) assays are the gold standard for the early diagnosis and risk stratification of acute myocardial infarction (AMI). PERFORM-TSIX (clinicaltrials.gov identifier: NCT06734117) is a prospective, international, observational, longitudinal cohort study to evaluate the clinical performance of the next-generation Elecsys® Troponin T hs Gen 6 assay; the study design is presented here.

**Objectives:**

The primary objective is to determine the sensitivity of the Troponin T hs Gen 6 assay for the detection of centrally adjudicated AMI diagnosis at 3 h post-emergency department (ED) presentation. Secondary objectives include evaluation of clinical performance at 0, 1-, 5-, and 6-h post-ED presentation and validation of thresholds for a 0/1-h algorithm to rule out AMI. Exploratory objectives include validation of thresholds for a 0/1-h algorithm to rule in AMI and a 0/2-h algorithm to rule in/out AMI and evaluation of prognostic performance at 30 and 180 days.

**Methods:**

PERFORM–TSIX enrolled 5631 participants across 50 sites from the USA, Europe, China, and Japan. Patients aged ≥ 20 years presenting to the ED with symptoms/signs of acute coronary syndrome were enrolled. All patients were required to have cTn measured as part of their routine care; AMI diagnosis was adjudicated by an independent clinical events committee in accordance with the Fourth Universal Definition of MI, blinded to the results of the Troponin T hs Gen 6 assay.

**Conclusion:**

PERFORM-TSIX will determine the clinical performance of the Troponin T hs Gen 6 assay for the diagnosis of AMI in a large, diverse global population.

**Graphical Abstract:**

**Figure:**
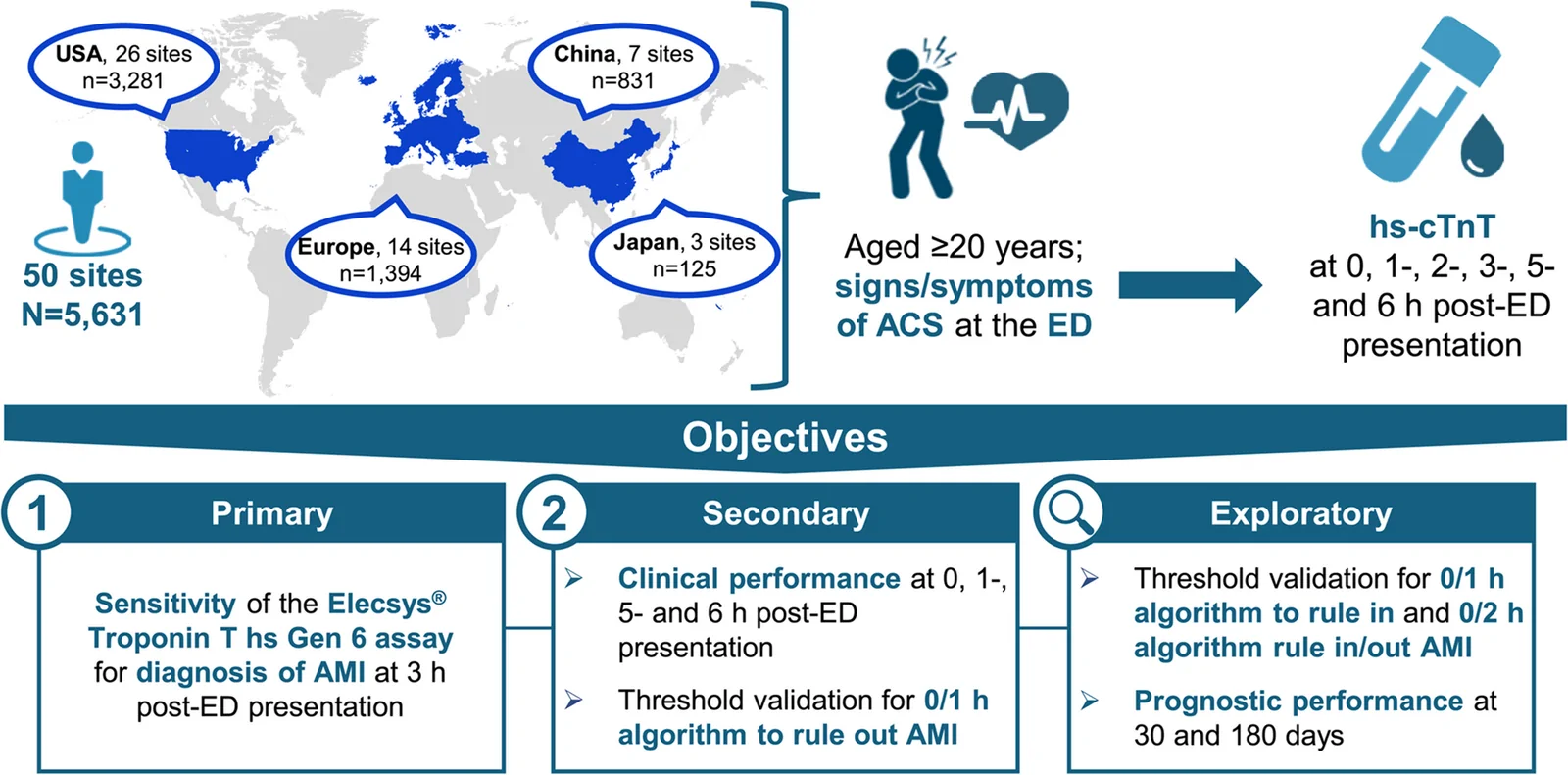

**Supplementary Information:**

The online version contains supplementary material available at https://doi.org/10.1007/s00392-025-02842-x.

## Introduction

Chest discomfort and other symptoms suggestive of acute myocardial infarction (AMI) are common reasons for patients to present to the emergency department (ED) [1]. In addition to clinical assessment and an electrocardiogram (ECG), measurements of cardiac troponin (cTn) are integral to the risk stratification and early diagnosis of AMI [2–6]. Diagnosis of AMI requires the detection of acute myocardial injury, defined as a rise and/or fall in cTn with at least one value above the sex-specific 99th percentile upper reference limit (URL), combined with clinical evidence of myocardial ischemia [5].

Clinical outcomes are dependent on a rapid and accurate diagnosis of AMI to ensure prompt treatment and coronary revascularization, as well as efficient disposition from the ED [7]. cTn measured by a high-sensitivity (hs) assay is considered the gold standard for the early risk stratification and diagnosis of AMI and is recommended by international guidelines [8–11]. The International Federation of Clinical Chemistry (IFCC) designates a cTn assay as hs when cTn is measurable above the lower limit of detection in more than half of all males and females in a healthy reference population, and the assay achieves a coefficient of variation (CV) of < 10% at the sex-specific 99th percentile URL [12, 13]. The introduction of hs-cTn assays allowed for the development of several accelerated algorithms, such as the European Society of Cardiology (ESC) 0/− 1 and − 2 h (h) algorithms, to support timely clinical decisions [8, 9, 14–18].

The next-generation Elecsys® Troponin T hs Gen 6 assay (Roche Diagnostics, Rotkreuz, Switzerland) has been developed using electrochemiluminescence technology to allow for rapid, quantitative *in vitro* determination of cTnT in serum and plasma. This assay has previously demonstrated higher analytical sensitivity and accuracy, especially at lower cTnT concentrations than earlier assay generations, as well as improved interference tolerance, most notably with respect to hemolysis [19]. This aims to optimize the performance of the assay for standard-of-care applications. Here, we present the design of PERFORM-TSIX, a prospective, international, observational, multicenter, longitudinal cohort study that aims to evaluate the clinical performance of the new Troponin T hs Gen 6 assay. The study is being conducted across multiple global regions in a representative, diverse population of patients presenting to the ED with suspected acute coronary syndrome (ACS), which may lead to a diagnosis of AMI.

## Methods

### Study design and study population

**Fig. 1:**
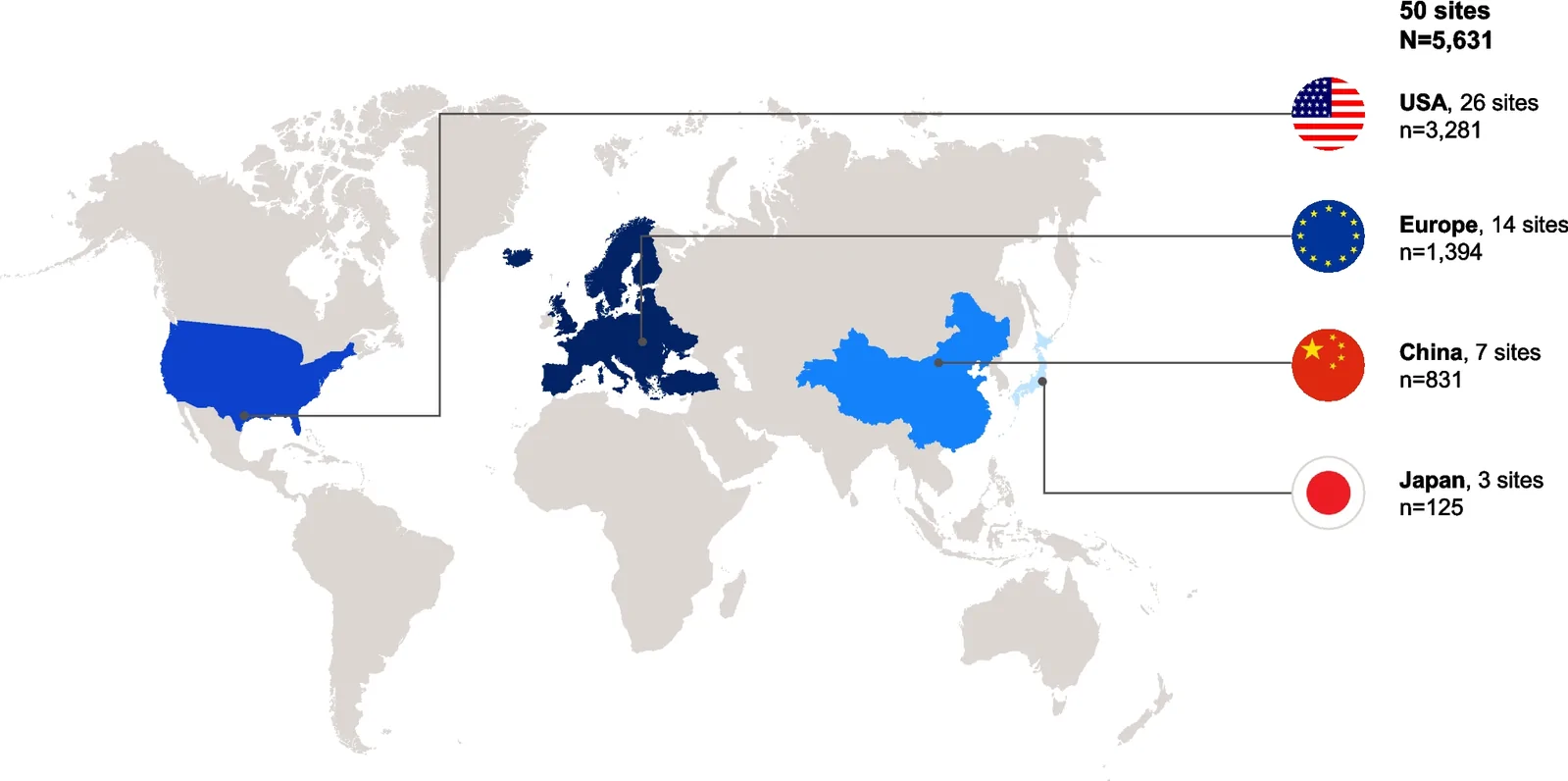
Map of study sites by region

The PERFORM-TSIX study (retrospectively registered at clinicaltrials.gov [NCT06734117, December 2024]) is a prospective, international, observational, multicenter, longitudinal cohort study that aimed to enroll 5600 patients with suspected ACS across 50 sites from the United States of America (USA), Europe, China, and Japan [20]. Details on study sites are shown in Fig. 1 and suppl. Table 1. Recruitment commenced in November 2021 and continued for 31.5 months. In total, 5631 participants were enrolled across the regions (US, *n* = 3281; Europe, *n* = 1394; China, *n* = 831; Japan, *n* = 125). The study population was an all-comers cohort consisting of adults aged ≥ 20 years who presented to the ED with symptoms or signs of possible ACS. All patients were required to have cTn or other cardiac markers (creatine-kinase-MB) measured as part of their routine care. Patients were eligible for inclusion if they demonstrated symptoms suggestive of ACS and/or myocardial ischemia: chest pain; pressure; or a burning sensation across the precordium and epigastrium; pain that radiates (to neck, shoulder, jaw, back, upper abdomen, or either arm); acute onset or worsening dyspnea, gastrointestinal symptoms (nausea, vomiting, or indigestion); lightheadedness or syncope; diaphoresis; generalized weakness or fatigue; or if patients were asymptomatic but AMI was suspected.

Patients who met the inclusion criteria provided written informed consent prior to enrollment. The study was conducted in accordance with Good Clinical Practice and the Declaration of Helsinki, and ethical approval was obtained at each site from the pertinent ethical review board. A steering committee with expertise in cardiovascular biomarkers and AMI governed the PERFORM–TSIX study, which is funded by F Hoffmann-La Roche.

### Objectives

Objectives and outcome measures are detailed in Table 1. The primary objective is to determine the clinical performance of the Troponin T hs Gen 6 assay for the diagnosis of AMI at 3 h post-ED presentation. Secondary objectives are to evaluate the clinical performance at additional time points (0, 1, 5, and 6 h) post-ED presentation and to validate thresholds for use in a 0/1-h algorithm to rule out AMI. Exploratory objectives are to validate thresholds for a 0/1-h algorithm to rule in AMI and a 0/2-h algorithm to rule in and rule out AMI and to evaluate the prognostic value of the Troponin T hs Gen 6 assay at 30 and 180 days.

**Table 1:** Summary of objectives and outcome measures for the PERFORM–TSIX study

	Objective	Outcome measure
Primary endpoint	• To determine the clinical performance of the Troponin T hs Gen 6 assay for the diagnosis of AMI at 3 h post-ED presentation using the previously determined universal and sex-specific 99th percentile URLs	• Sensitivity of the Troponin T hs Gen 6 assay for a CEC-adjudicated diagnosis of AMI when measured 3 h post-ED presentation (defined as samples taken 121–240 min post-ED presentation) • Acceptance criterion: > 81% for the LCL for sensitivity
Secondary endpoints	• To determine the clinical performance of the Troponin T hs Gen 6 assay additional time points using the previously determined universal and sex-specific a 99th percentile URLs	• Sensitivity, specificity, NPV and PPV of the Troponin T hs Gen 6 assay at additional time points (0 h [samples taken 0 to 60 min post-ED presentation], 1 h [samples taken 61 to 120 min post-ED presentation], 5 h [samples taken 241 to 360 min post-ED presentation] and 6 h [samples taken > 360 min post-ED presentation])
	• To validate thresholds for use in a 0/1-h algorithm to rule out AMI using the Troponin T hs Gen 6 assay	• NPV of the Troponin T hs Gen 6 assay to rule out AMI using a 0/1-h algorithm (0 h [samples taken 0 to 60 min post-ED presentation]/1 h [samples taken 30 to 90 min post-ED presentation]) • Acceptance criterion: > 98% for the LCL for NPV
Exploratory endpoints	• To validate thresholds for a 0/1-h algorithm to rule in AMI in patients presenting to the ED using the Troponin T hs Gen 6 assay	• Sensitivity, specificity, PPV and NPV of the Troponin T hs Gen 6 assay to rule in AMI using a 0/1-h algorithm (0 h [samples taken 0 to 60 min post-ED presentation]/1 h [samples taken 30 to 90 min post-ED presentation])
	• To validate a 0/2-h algorithm to rule in and rule out AMI in patients presenting to the ED using the Troponin T hs Gen 6 assay	• Sensitivity, specificity, PPV and NPV of the Troponin T hs Gen 6 assay to rule in and rule out AMI using a 0/2-h algorithm (0 h [samples taken 0 to 60 min post-ED presentation]/2 h [samples taken 91 to 150 min post-ED presentation])
	• To evaluate the prognostic value of the Troponin T hs Gen 6 assay at 30 and 180 days	• Risk prediction for MACE at 30 and 180 days for the different algorithmic outcome categories (rule-out, observe, rule-in)

*AMI* acute myocardial infarction, *CEC* clinical events committee, *ED* emergency department, *LCL* lower confidence limit, *MACE* major adverse cardiovascular outcomes, *NPV* negative predictive value, *PPV* positive predictive value, *URL* upper reference limit

### Outcomes

The primary diagnostic outcome measure is the sensitivity of the Troponin T hs Gen 6 assay for a clinical events committee (CEC)–adjudicated diagnosis of AMI when measured 3 h post-ED presentation (defined as samples collected 121 to 240 min post-ED presentation). Patients are only eligible for inclusion in the evaluation of the primary diagnostic outcome if at least one blood sample was collected within the defined timeframe of 121 to 240 min post-ED presentation. If more than one sample was collected within the window, the sample closest to the middle of the window will be used. Acceptance criterion for the primary endpoint is 81% for the lower confidence limit (LCL) for sensitivity.

Secondary outcome measures for the clinical performance of the Troponin T hs Gen 6 assay at additional time points are sensitivity, specificity, negative predictive value (NPV), and positive predictive value (PPV); measures to validate the thresholds for a 0/1-h algorithm to rule out AMI include sensitivity, specificity, NPV, and PPV. Acceptance criterion for the 0/1-h algorithm to rule out AMI is 98% for the LCL for NPV.

Exploratory outcome measures to validate the thresholds for a 0/1-h algorithm to rule in AMI and a 0/2-h algorithm to rule in and rule out AMI include sensitivity, specificity, NPV, and PPV.

To evaluate the prognostic value of the Troponin T hs Gen 6 assay, risk prediction for major adverse cardiovascular events (MACE) at 30 and 180 days will be analyzed for the different algorithmic outcome categories (rule-out, observe, rule-in).

### Study procedures

#### Sample collection, storage, and measurement

**Fig. 2:**
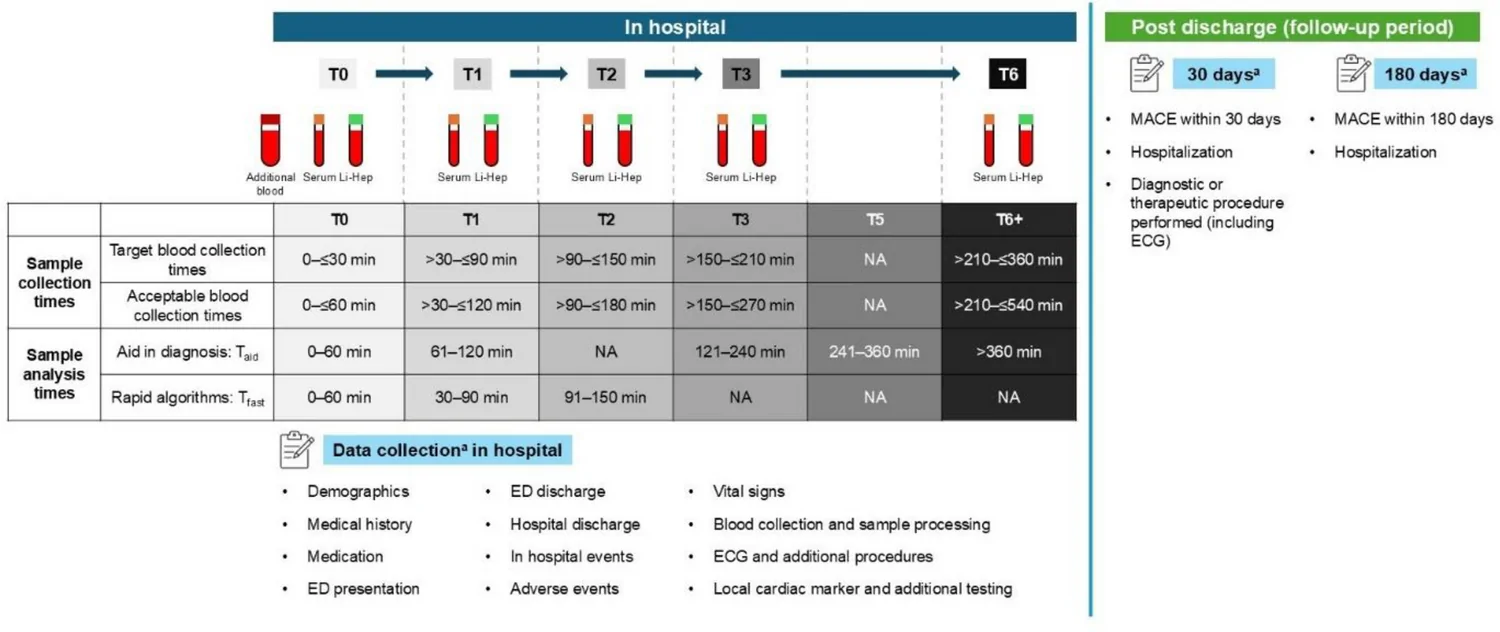
Schematic diagram of the PERFORM–TSIX study design. ^a^Please see suppl. Table 3 for details

Venous blood samples were collected in serum and lithium-heparin (LiHep) plasma tubes within 1 h, and ideally within 30 min, of presentation to the ED (defined as time point [T]0). T0 was defined as registration in the ED; if patients arrived by ambulance and registration was not performed, triage time or the earliest time stamp available in the electronic case report form (eCRF) was used. Further samples were collected at 1, 2, and 3 h (T1–3) and up to 6 h (T6) post-ED presentation; target and acceptable blood collection times are detailed in Fig. 2. All blood samples were processed into serum and plasma and centrifuged in accordance with the guidelines of the sample tube manufacturers. Samples were then frozen at − 20 °C or colder within 4 h of blood draw. All samples were visually inspected prior to the experimental run to ensure no clots, precipitates, foam, or droplets on the container wall were present.

In the USA, Europe, and Japan, cTn in serum and LiHep plasma samples collected at the study sites were measured at one of five core laboratories, using the Troponin T hs Gen 6 assay on the Cobas® e 801 analyzer. In China, serum and LiHep plasma samples were measured at collection sites using the Troponin T hs Gen 6 assay on the Cobas e 801 analyzer. Details on testing sites are shown in suppl. Table 2. All samples were also measured using the current assay generation, Elecsys Troponin T (US)/Elecsys Troponin T hs Biotin PU STAT (rest of world) on the Cobas e 801 analyzer.

In addition to measuring cTn, multiple pathophysiological markers were measured to gather further information on comorbidities and to detect possible analytical interferences. Across all regions, a blood sample was used to measure N-terminal pro B-type natriuretic peptide (NT-proBNP), creatinine, and serum indices (hemolysis, icterus, and lipemia). In the USA, Europe, and Japan, bilirubin and triglycerides were measured from blood samples taken at each time point (results for individual time points will be excluded if out of range), whereas in China, levels were only measured once and will be applied to all time points. Ranges for exclusion were serum hemolysis index > 1000 (> 100 when using Elecsys Troponin T hs Biotin PU STAT assay); bilirubin > 25 mg/dL; or triglycerides > 1500 mg/dL; all tested at the testing site. Additional serum and plasma were retained for future use in other ethically approved studies.

#### Data collection

Data were collected and recorded in eCRFs and included patient demographics, vital signs, medical history, medications, electrocardiography, additional cardiac investigations, other routine laboratory testing, length of hospital stay, discharge/admission decision, any in-hospital cardiac events, and the final clinical diagnosis as recorded by sites. The full list of parameters that were collected is shown in suppl. Table 3. For adverse event (AE) and serious adverse event (SAE) reporting, the investigator determined if any AE/SAE occurred, and all study-related SAEs/AEs were recorded in the eCRF. SAEs were reported within 3 days of knowledge of the event.

#### Adjudication of the diagnosis of AMI

The diagnosis of AMI (type 1, 2, 4b, 4c) was adjudicated by a central and independent CEC in accordance with the Fourth Universal Definition of MI [5], blinded to the results of the Troponin T hs Gen 6 assay. AMI was defined as a rise and/or fall in cTn with at least one value above the 99th percentile URL, combined with clinical evidence of myocardial ischemia [5]. Each case was reviewed independently by two clinicians blinded to each other’s determination. If the reviewers’ diagnoses were the same, the diagnosis was finalized; if the reviewers’ diagnoses differed, a third clinician reviewed the presentation to establish the final adjudicated diagnosis. Each adjudicator had access to de-identified clinical data, including a description of symptoms, time of symptom onset, all 12-lead ECGs from the ED, echocardiography, cardiac stress testing and imaging, results of the local site cTn (including manufacturer and assay used, time of blood draw, result value and cut-offs used locally) and/or other cardiac biomarker tests, specified laboratory tests, treatment and hospital admission and discharge information. Results of the centrally measured current clinically available Elecsys Troponin T hs assay, NT-proBNP measurement and 30-day follow-up were also provided to the adjudicators. Based on all available clinical information, diagnosis was adjudicated in line with the Fourth Universal Definition of MI and classified into the following categories: (1) AMI (non-ST-elevation myocardial infarction (NSTEMI), ST-segment elevation myocardial infarction (STEMI), ECG not available or interpretable); (2) unstable angina; (3) cardiovascular non-coronary artery disease; (4) non-cardiac disease or symptoms of unknown origin. The clinical performance of the universal 99th percentile URL of the Troponin T hs Gen 6 assay was analyzed based on adjudication using the universal (non-sex-specific) cut-off for the current assay generation (Elecsys Troponin T hs) and performance of sex-specific 99th percentile URLs derived for the Troponin T hs Gen 6 assay were analyzed based on adjudication using sex-specific cut-offs. This was the gold standard diagnosis used to determine the clinical performance of the Troponin T hs Gen 6 assay.

#### Patient follow-up and outcomes

Patient follow-ups were conducted at 30 (+7) and 180 (+7) days post-ED presentation and consisted of a thorough chart review followed by a phone interview. To enhance the completeness of follow-up, patient calls were attempted on at least three different occasions. If approved by ethics committees, sites could also send a letter to the patient, and a follow-up reminder card could be provided at the time of enrollment.

Subsequent hospitalization for any cause, MACE (MI (including type of MI for the 30-day follow-up), all-cause death (cardiac and non-cardiac) [21]) occurring following discharge, and lost to follow-up rates were recorded in the eCRF (suppl. Table 3) for 30- and 180-day outcomes. Risk stratification and prognostic value of the Troponin T hs Gen 6 assay will be evaluated. For the 30-day follow-up only, any diagnostic or therapeutic procedures that occurred since discharge and the date they were performed (including ECG, echocardiogram, cardiac computed tomography scan, cardiac magnetic resonance imaging (MRI), stress tests, invasive angiography, percutaneous transluminal coronary angioplasty (urgent/routine), and coronary artery bypass graft (urgent/routine)) were recorded in the eCRF (suppl. Table 3).

### Statistical analysis

Baseline characteristics, including distributions of age, sex, ethnicity, and race, will be summarized as proportions, mean and standard deviation, or median and inter-quartile range as appropriate in the study population, with stratification according to whether the final adjudicated diagnosis was AMI.

The universal and sex-specific 99th percentile URLs derived from a separate reference range study (REF-TSIX) will be used to classify the Troponin T hs Gen 6 assay results, with values above indicative of a potential AMI [5]. Sensitivity, specificity, NPV, and PPV will be calculated using cTnT measurements taken at sample analysis times T0, T1, T3, T5, and T6 (Fig. 2), based on all evaluable samples available at each time point. Clinical performance measures will be reported with 95% confidence intervals (CIs) estimated using the Wilson score method [22]. The results will be shown for the total cohort as well as separately for females and males (determined by sex assigned at birth). A subpopulation excluding STEMI patients will also be analyzed.

Thresholds and delta change values for rapid algorithms (0/1 h and 0/2 h) to rule out and rule in AMI for evaluation in this study will be determined in a separate cohort, independent of this analysis. The results of the independent cohort analysis will be validated in this PERFORM-TSIX study cohort. Sensitivity, specificity, PPV, and NPV will be calculated with 95% CIs for the rule-out and rule-in criteria.

Risk prediction based on Troponin T hs Gen 6 strata and prognostic value for the classification of the algorithmic outcome categories (rule-out zone, observe zone, rule-in zone) will be analyzed with respect to short-term (30-day) and long-term (180-day) outcomes (MACE [MI (including type of MI for the 30-day follow-up), all-cause death (cardiac and non-cardiac) [21]). The Kaplan–Meier method will be used to perform time-to-event analysis to compare the survival probabilities for different TnT strata and algorithmic outcome categories (rule-out zone, observe zone, rule-in zone). To this end, hazard ratios calculated from the Cox regression model and cumulative event-free rates will be reported to quantify the difference in risk between groups.

To determine the clinical performance of the Troponin T hs Gen 6 assay measured 3 h following presentation to the ED (primary objective), the sensitivity for a CEC-adjudicated diagnosis of AMI will be calculated at the sex-specific and universal 99th percentile URL. Assuming an expected sensitivity of 90%, and setting the two-sided significance level of 5%, a sample size of at least 160 patients with AMI is required to give power of at least 80% [23], using a statistical test based on an acceptance criterion of 81% for the LCL for sensitivity.

To validate the clinical performance of the Troponin T hs Gen 6 assay in a 0/1 h algorithm (secondary objective), an NPV of greater than 99% was expected for those patients stratified as rule out [8]. To provide at least 80% power at 5% significance, 1356 patients were required who met the criteria for rule out, using a statistical test based on the acceptance criterion of 98% for the lower bound of the 95% CI for the NPV. Assuming approximately 60% of patients met the criteria for rule out [4, 24], a total sample size of 2326 evaluable patients with samples at 0 and 1 h was required. Such a total sample size enabled the certainty of 95% that 1356 patients who met the criteria for rule out would be found in a sample of 2326 patients (Schaetzkin adjustment) [25], given the assumptions mentioned above. Based on a minimum sample size of 2326 patients and accounting for incomplete sample collection for T0/T1 samples of around 35% [23], a 10% dropout rate, as well as regional representation due to regulatory requirements, we aimed to recruit approximately 5600 patients.

All cTn results will be stored in a Web-based Computer Aided Evaluation (WebCAEv) database. For statistical analysis, the data from WebCAEv and the eCRF will be exported and merged using the statistics software R [26].

## Discussion

The PERFORM–TSIX study aims to evaluate the clinical performance of the Troponin T hs Gen 6 assay as an aid in the diagnosis of AMI in a prospective, multinational real-world cohort. Clearly defined acceptance thresholds and the use of diagnostic, algorithmic validation, and prognostic endpoints will ensure the study results are relevant for clinical practice. The patient enrollment using broad inclusion criteria and geographically diverse sites will reflect the real-world use of cTn assays. The inclusion criteria include secondary symptoms of AMI, which are more prevalent in numerous subcohorts, including elderly patients and patients with diabetes [27, 28]. While female and male patients are as likely to present with chest pain, female patients are more likely to report additional symptoms [29–31]. The inclusion of symptoms other than chest pain should ensure that the results are relevant to the broad range of patients who undergo evaluation for suspected ACS. In addition, the adjudicated final diagnosis is based on all available clinical data, irrespective of the number of cTn time points available.

The global population, large sample size, and representation from different healthcare settings, including community hospitals and tertiary care centers, as well as liberal inclusion criteria, will enhance the real-world applicability of the study results. Notably, previous studies evaluating the performance of hs-cTn assays have tended to be performed in smaller populations enrolled from a single healthcare system or country [3, 32–36]. The large sample size will also allow for secondary analyses, while the inclusion of patients from different racial and ethnic groups will enable the study to account for potential variations in cTn levels based on race and ethnicity [37, 38].

Validation of the Troponin T hs Gen 6 assay for use in rapid rule-in and rule-out algorithms is an added benefit of the study design and will allow the assessment of the assay performance in guideline-recommended clinical pathways. In a meta-analysis of 30,066 patients, the 0/1- and 0/2-h algorithms demonstrated a higher sensitivity and NPV than a 0/3-h algorithm using the 99th percentile to rule in and rule out AMI [39]. Additionally, it has been shown that the ESC 0/1-h algorithm allows earlier clinical decision-making, which can reduce the total time to discharge by reducing the length of stay in the ED [3, 7, 39, 40]. Validating the performance of the new assay in these rapid algorithms will help to support effective implementation of the Troponin T hs Gen 6 assay.

In addition to diagnosing AMI when a patient presents to the ED, cTn may be beneficial for the prognosis of other cardiovascular diseases [41–43]. As well as determining the diagnostic use of the Troponin T hs Gen 6 assay, the PERFORM-TSIX study will evaluate the assay’s utility for risk stratification and prediction of subsequent death and MACE at 30 and 180 days.

Our study is not without limitations. First, while a global study provides a robust evaluation of overall assay performance, because no single population is solely represented, application of the overall results to any specific locality may require subset analysis for the best accuracy. It is also possible that local or regional standards of care or practice protocols at individual hospitals influenced sample collection procedures and the availability of samples at some time points. In addition, as with any prospective investigation, patients may be lost to follow-up; however, the large sample size and robust follow-up methods (e.g., second telephone number, checking hospital records, multiple phone calls, and sending a letter to the patient’s address) will improve follow-up completion and reduce the risk of selection bias. Furthermore, patients who missed any of the predefined sample collection time points were excluded from the analysis at that time point, reducing the number of samples available for analysis and providing undefined bias. However, it might be noted that patients missing later follow-up time points are likely to be lower-risk patients (i.e., those with early or rapid discharge) and the exclusion of these patients is unlikely to have a significant influence on our outcomes. Those patients still received an adjudicated diagnosis.

As our study included an all-comers population, it is possible that patients may have been enrolled late after the onset of symptoms; therefore, a subanalysis based on early versus late presenters according to symptom onset (rather than presentation to the ED) may be beneficial. In addition, the all-comers population will result in the inclusion of patients with STEMI, which may bias the performance of a cTn assay as patients with STEMI show higher cTn levels compared with patients with NSTEMI [44]. Despite higher cTn concentrations in STEMI overall, a high proportion of patients with STEMI have low or undetectable cTn concentrations at presentation due to both early presentation as well as abrupt coronary occlusion that may initially prevent cTn release into the circulation [45], which could lead to false-negative results. cTn is not the recommended way to diagnose STEMI in the ED, where time is of the essence and the ECG and clinical presentation alone are typically used. However, patients with STEMI should not be actively excluded in trials evaluating cTn assays, as ST elevations may be unclear in the initial ECG and in such settings, cTn can be important to confirm a diagnosis of AMI. To overcome this potential bias, a subanalysis excluding STEMI patients will also be performed.

Finally, as cTn is integral to the diagnosis of AMI, there is a risk of confirmation bias, but this is integral to all diagnostic performance studies evaluating novel cTn assays. It may be speculated that if a different hs-cTn assay were used as the gold standard, the data to support clinical performance may vary. However, an effort has been made to minimize this by using an independent CEC to perform diagnostic adjudication using standardized criteria, including rules about what to do when some of the clinical and/or biochemical information was missing, whilst blinded to the results of the Troponin T hs Gen 6 assay.

## Conclusion

PERFORM-TSIX is a prospective, international, observational, multicenter, longitudinal cohort study evaluating the clinical performance of the Troponin T hs Gen 6 assay as an aid in the diagnosis of AMI. The study will also determine the assay’s performance to rule in or rule out AMI using rapid algorithms and to risk-stratify patients for short- and long-term outcomes. The main strengths of the study are the large sample size, diverse global population, and inclusion of healthcare centers with different levels of care, which will ensure the findings can be applied in many populations and settings.

## Supplementary Information

Below is the link to the electronic supplementary material.

ESM1 (DOCX 53.3 KB)

## Data Availability

Requests concerning the data supporting the findings of this study can be directed to rotkreuz.datasharingrequests@roche.com for consideration.

## Declarations

### Disclaimer

ELECSYS and COBAS are trademarks of Roche.

All other product names and trademarks are the property of their respective owners.

## References

[1] Stepinska J, Lettino M, Ahrens I et al (2020) Diagnosis and risk stratification of chest pain patients in the emergency department: focus on acute coronary syndromes. A position paper of the Acute Cardiovascular Care Association. Eur Heart J 9:76–89. 10.1177/2048872619885346 31958018

[2] Arslan M, Dedic A, Boersma E, Dubois EA (2020) Serial high-sensitivity cardiac Troponin T measurements to rule out acute myocardial infarction and a single high baseline measurement for swift rule-in: a systematic review and meta-analysis. Eur Heart J Acute Cardiovasc Care 9:14–22. 10.1177/2048872618819421 30618277 PMC7008551

[3] Chapman AR, Fujisawa T, Lee KK et al (2019) Novel high-sensitivity cardiac troponin I assay in patients with suspected acute coronary syndrome. Heart 105:616. 10.1136/heartjnl-2018-314093 30442743 PMC6580754

[4] Reichlin T, Schindler C, Drexler B et al (2012) One-hour rule-out and rule-in of acute myocardial infarction using high-sensitivity cardiac Troponin T. Arch Intern Med 172:1211–1218. 10.1001/archinternmed.2012.3698 22892889

[5] Thygesen K, Alpert JS, Jaffe AS et al (2018) Fourth universal definition of myocardial infarction (2018). J Am Coll Cardiol 72:2231–2264. 10.1016/j.jacc.2018.08.1038 30153967

[6] Westermann D, Neumann JT, Sörensen NA, Blankenberg S (2017) High-sensitivity assays for troponin in patients with cardiac disease. Nat Rev Cardiol 14:472–483. 10.1038/nrcardio.2017.48 28383022

[7] Lee CC, Huang SS, Yeo YH et al (2020) High-sensitivity-cardiac troponin for accelerated diagnosis of acute myocardial infarction: a systematic review and meta-analysis. Am J Emerg Med 38:1402–1407. 10.1016/j.ajem.2019.11.035 31932131

[8] Byrne RA, Rossello X, Coughlan JJ et al (2023) 2023 ESC guidelines for the management of acute coronary syndromes: developed by the task force on the management of acute coronary syndromes of the European Society of Cardiology (ESC). Eur Heart J 44:3720–3826. 10.1093/eurheartj/ehad191 37622654

[9] Sandoval Y, Apple FS, Mahler SA, Body R, Collinson PO, Jaffe AS (2022) High-sensitivity cardiac troponin and the 2021 AHA/ACC/ASE/CHEST/SAEM/SCCT/SCMR guidelines for the evaluation and diagnosis of acute chest pain. Circulation 146:569–581. 10.1161/circulationaha.122.059678 35775423

[10] Tan WCJ, Inoue K, AbdelWareth L et al (2020) The Asia-Pacific Society of Cardiology (APSC) expert committee consensus recommendations for assessment of suspected acute coronary syndrome using high-sensitivity cardiac Troponin T in the emergency department. Circ J 84:136–143. 10.1253/circj.CJ-19-0874 31852863

[11] McGrath S, Alaour B, Kampourakis T, Marber M (2025) Cardiac troponin: fragments of the future? JACC Adv 4:101695. 10.1016/j.jacadv.2025.101695 40286361 PMC12102503

[12] Aakre KM, Saenger AK, Body R et al (2022) Analytical considerations in deriving 99th percentile upper reference limits for high-sensitivity cardiac troponin assays: educational recommendations from the IFCC committee on clinical application of cardiac bio-markers. Clin Chem 68:1022–1030. 10.1093/clinchem/hvac092 35716089

[13] Wu AHB, Christenson RH, Greene DN et al (2018) Clinical laboratory practice recommendations for the use of cardiac troponin in acute coronary syndrome: expert opinion from the Academy of the American Association for Clinical Chemistry and the Task Force on Clinical Applications of Cardiac Bio-Markers of the International Federation of Clinical Chemistry and Laboratory Medicine. Clin Chem 64:645–655. 10.1373/clinchem.2017.277186 29343532

[14] Collet JP, Thiele H, Barbato E et al (2021) 2020 ESC guidelines for the management of acute coronary syndromes in patients presenting without persistent ST-segment elevation. Eur Heart J 42:1289–1367. 10.1093/eurheartj/ehaa575 32860058

[15] Gulati M, Levy PD, Mukherjee D et al (2022) 2021 AHA/ACC/ASE/CHEST/SAEM/SCCT/SCMR guideline for the evaluation and diagnosis of chest pain: a report of the American College of Cardiology/American Heart Association Joint Committee on clinical practice guidelines. J Cardiovasc Comput Tomogr 16:54–122. 10.1016/j.jcct.2021.11.009 34955448

[16] Rao SV, O’Donoghue ML, Ruel M et al (2025) 2025 ACC/AHA/ACEP/NAEMSP/SCAI guideline for the management of patients with acute coronary syndromes: a report of the American College of Cardiology/American Heart Association Joint Committee on clinical practice guidelines. Circulation 151:e771–e862. 10.1161/cir.0000000000001309 40014670

[17] Sandoval Y, Lewis BR, Mehta RA et al (2022) Rapid exclusion of acute myocardial injury and infarction with a single high-sensitivity cardiac Troponin T in the emergency department: a multicenter United States evaluation. Circulation 145:1708–1719. 10.1161/circulationaha.122.059235 35535607 PMC9568367

[18] Sandoval Y, Jaffe AS (2017) Using high-sensitivity cardiac Troponin T for acute cardiac care. Am J Med 130:1358-1365.e1351. 10.1016/j.amjmed.2017.07.033 28843652

[19] Knoll M DL, Mueller C, Rösser AFA, Kurtoic D, Wahl A, Mills NL, Giannitsis E, Peacock WF, Meex SJR (18–22 May 2025) Analytical performance of a Troponin T high-sensitivity Gen 6 assay. EuroMedLab; Brussels, Belgium.

[20] NCT06734117. A study of Elecsys® Troponin T hs Gen 6 in participants with symptoms of acute coronary syndrome (PERFORM-TSIX). https://clinicaltrials.gov/study/NCT06734117. Accessed 15 August 2025

[21] Hicks KA, Mahaffey KW, Mehran R et al (2018) 2017 cardiovascular and stroke endpoint definitions for clinical trials. J Am Coll Cardiol 71:1021–1034. 10.1016/j.jacc.2017.12.048 29495982

[22] E.B W (1927) Probable inference, the law of succession, and statistical inference. J Am Stat Assoc 22:209–212. 10.2307/2276774

[23] Christenson RH, Duh SH, Mullins KE, LeClair MM, Grigorov IL, Peacock WF (2020) Analytical and clinical characterization of a novel high-sensitivity cardiac troponin assay in a United States population. Clin Biochem 83:28–36. 10.1016/j.clinbiochem.2020.05.014 32485169

[24] Mueller C, Giannitsis E, Christ M et al (2016) Multicenter evaluation of a 0-hour/1-hour algorithm in the diagnosis of myocardial infarction with high-sensitivity cardiac Troponin T. Ann Emerg Med 68:76-87.e74. 10.1016/j.annemergmed.2015.11.013 26794254

[25] Pepe MS (2003) The statistical evaluation of medical tests for classification and prediction. Oxford Statistical Science Series, Oxford University Press

[26] R Core Team (2018) The R project for statistical computing. https://www.R-project.org. Accessed 15 Aug 2025

[27] Canto JG, Shlipak MG, Rogers WJ et al (2000) Prevalence, clinical characteristics, and mortality among patients with myocardial infarction presenting without chest pain. JAMA 283:3223–3229. 10.1001/jama.283.24.3223 10866870

[28] Schmitz T, Wein B, Raake P et al (2024) Do patients with diabetes with new onset acute myocardial infarction present with different symptoms than non-diabetic patients? Front Cardiovasc Med 11:1324451. 10.3389/fcvm.2024.1324451 38287984 PMC10822885

[29] Coventry LL, Finn J, Bremner AP (2011) Sex differences in symptom presentation in acute myocardial infarction: a systematic review and meta-analysis. Heart Lung 40:477–491. 10.1016/j.hrtlng.2011.05.001 22000678

[30] Lichtman JH, Leifheit EC, Safdar B et al (2018) Sex differences in the presentation and perception of symptoms among young patients with myocardial infarction: evidence from the VIRGO study (variation in recovery: role of gender on outcomes of young AMI patients). Circulation 137:781–790. 10.1161/circulationaha.117.031650 29459463 PMC5822747

[31] Schulte KJ, Mayrovitz HN (2023) Myocardial infarction signs and symptoms: females vs. males. Cureus 15:e37522. 10.7759/cureus.37522 37193476 PMC10182740

[32] Kim S, Yoo SJ, Kim J (2020) Evaluation of the new Beckman Coulter Access hsTnI: 99th percentile upper reference limits according to age and sex in the Korean population. Clin Biochem 79:48–53. 10.1016/j.clinbiochem.2020.02.005 32059836

[33] Krintus M, Kozinski M, Boudry P et al (2014) European multicenter analytical evaluation of the Abbott ARCHITECT STAT high sensitive troponin I immunoassay. Clin Chem Lab Med 52:1657–1665. 10.1515/cclm-2014-0107 24897400

[34] Meek R, Cullen L, Lu Z, Nasis A, Kuhn L, Sorace L (2023) Suspected myocardial infarction in the emergency department: an evaluation of clinical thresholds for the Beckman Coulter Access hsTnI high-sensitivity cardiac troponin I assay. Emerg Med Australas 35:1005–1012. 10.1111/1742-6723.14282 37442553

[35] Pretorius CJ, Tate JR, Wilgen U, Cullen L, Ungerer JPJ (2018) A critical evaluation of the Beckman Coulter Access hsTnI: analytical performance, reference interval and concordance. Clin Biochem 55:49–55. 10.1016/j.clinbiochem.2018.03.003 29524431

[36] Sörensen NA, Goßling A, Neumann JT et al (2021) Diagnostic validation of a high-sensitivity cardiac troponin I assay. Clin Chem 67:1230–1239. 10.1093/clinchem/hvab070 34254126

[37] Gore MO, Seliger SL, Defilippi CR et al (2014) Age- and sex-dependent upper reference limits for the high-sensitivity cardiac Troponin T assay. J Am Coll Cardiol 63:1441–1448. 10.1016/j.jacc.2013.12.032 24530665 PMC3984900

[38] Kalaria TR, Harris N, Sensi H et al (2021) High-sensitivity cardiac troponin I: is ethnicity relevant? J Clin Pathol 74:709. 10.1136/jclinpath-2020-206951 33782194

[39] Chiang CH, Chiang CH, Pickering JW et al (2022) Performance of the European Society of Cardiology 0/1-hour, 0/2-hour, and 0/3-hour algorithms for rapid triage of acute myocardial infarction: an international collaborative meta-analysis. Ann Intern Med 175:101–113. 10.7326/m21-1499 34807719

[40] Bevins NJ, Chae H, Hubbard JA et al (2022) Emergency department management of chest pain with a high-sensitivity troponin-enabled 0/1-hour rule-out algorithm. Am J Clin Pathol 157:774–780. 10.1093/ajcp/aqab192 34893795 PMC9071328

[41] Roos A, Edgren G, Holzmann MJ (2022) Temporal changes of stable high-sensitivity cardiac Troponin T levels and prognosis. J Am Heart Assoc 11:e025082. 10.1161/jaha.121.025082 35621209 PMC9238698

[42] Vrsalovic M, Vrsalovic Presecki A, Aboyans V (2022) Cardiac troponins predict mortality and cardiovascular outcomes in patients with peripheral artery disease: a systematic review and meta-analysis of adjusted observational studies. Clin Cardiol 45:198–204. 10.1002/clc.23776 35132665 PMC8860477

[43] Omland T, Pfeffer MA, Solomon SD et al (2013) Prognostic value of cardiac troponin I measured with a highly sensitive assay in patients with stable coronary artery disease. J Am Coll Cardiol 61:1240–1249. 10.1016/j.jacc.2012.12.026 23414791

[44] Chin CT, Wang TY, Li S et al (2012) Comparison of the prognostic value of peak creatine kinase-MB and troponin levels among patients with acute myocardial infarction: a report from the Acute Coronary Treatment and Intervention Outcomes Network Registry-get with the guidelines. Clin Cardiol 35:424–429. 10.1002/clc.21980 22434769 PMC6652484

[45] Wereski R, Chapman AR, Lee KK et al (2020) High-sensitivity cardiac troponin concentrations at presentation in patients with ST-segment elevation myocardial infarction. JAMA Cardiol 5:1302–1304. 10.1001/jamacardio.2020.2867 32785613 PMC7675101

