# Publisher Correction: Clinical performance of the next generation Elecsys Troponin T high-sensitivity Gen 6 assay in acute coronary syndrome (PERFORM-TSIX): study design

**DOI:** 10.1007/s00392-026-02969-5

**Published:** 2026-07-27

**Authors:** Lori B. Daniels, Evangelos Giannitsis, Christian Mueller, Steven J. R. Meex, David Buehlmann, Dunja Kurtoic, Garnet Bendig, Mette Cole, Richard Body, Robert H. Christenson, Christa Cobbaert, Christopher R. deFilippi, Kai M. Eggers, Kenji Inoue, Allan S. Jaffe, Cian P. McCarthy, James McCord, Johannes T. Neumann, Torbjørn Omland, Cynthia Papendick, Yader Sandoval, Jack Wei Chieh Tan, Martin P. Than, Raphael Twerenbold, Nicholas L. Mills, W. Frank Peacock

**Affiliations:** 1 Division of Cardiovascular Medicine, Department of Medicine, University of California, La Jolla, San Diego, CA USA; 2 Department of Internal Medicine III, Cardiology University Hospital of Heidelberg, Heidelberg, Germany; 3 Cardiovascular Research Institute Basel (CRIB) and Department of Cardiology, University Hospital Basel, University of Basel, Basel, Switzerland; 4 Central Diagnostic Laboratory, Maastricht University Medical Center, Maastricht, The Netherlands; 5 CARIM School for Cardiovascular Diseases, Maastricht University, Maastricht, The Netherlands; 6 Clinical Development, Roche Diagnostics International AG, Rotkreuz, Switzerland; 7 Biostatistics, Roche Diagnostics International AG, Rotkreuz, Switzerland; 8 Clinical Operations, Roche Diagnostics GmbH, Penzberg, Germany; 9 Clinical Operations, Roche Molecular Systems, Inc., Indianapolis, IN USA; 10 Emergency Department, Manchester University NHS Foundation Trust, Manchester Academic Health Science Centre, Manchester, UK; 11 Division of Cardiovascular Sciences, University of Manchester, Manchester, UK; 12 Department of Pathology, University of Maryland School of Medicine, Baltimore, MD USA; 13 Department of Clinical Chemistry and Laboratory Medicine, Leiden University Medical Center, Leiden, The Netherlands; 14 Departments of Medicine and Pathology, University of Maryland School of Medicine, Baltimore, MD USA; 15 Department of Medical Sciences, Uppsala University, Uppsala, Sweden; 16 Tokyo Heart Rhythm Clinic Shinjuku, Tokyo, Japan; 17 Department of Cardiovascular Biology and Medicine, Juntendo University Graduate School of Medicine, Tokyo, Japan; 18 Department of Cardiovascular Medicine and Department of Laboratory Medicine and Pathology, Mayo Clinic, Rochester, MN USA; 19 Heart and Vascular Institute, Massachusetts General Brigham and Harvard Medical School, Boston, MA USA; 20 Heart and Vascular Institute, Henry Ford Health, Detroit, MI USA; 21 Department of Cardiology, University Heart and Vascular Centre Hamburg, University Medical Center Hamburg-Eppendorf, Hamburg, Germany; 22 Center for Population Health Innovation, University Heart and Vascular Center Hamburg, University Medical Center Hamburg-Eppendorf, Hamburg, Germany; 23 German Center for Cardiovascular Research, Partner Site Hamburg/Kiel/Lübeck, Hamburg, Germany; 24 Department of Epidemiology and Preventive Medicine, School of Public Health and Preventive Medicine, Monash University, Melbourne, Australia; 25 Department of Cardiology, Akershus University Hospital, Lørenskog, Norway; 26 Institute of Clinical Medicine, K. G. Jebsen Centre for Cardiac Biomarkers, University of Oslo, Oslo, Norway; 27 Department of Emergency Medicine, The Royal Adelaide Hospital, Central Adelaide Local Health Network, Adelaide, South Australia Australia; 28 Adelaide Medical School, The University of Adelaide, Adelaide, South Australia Australia; 29 Minneapolis Heart Institute, Abbott Northwestern Hospital and Center for Coronary Artery Disease, Minneapolis Heart Institute Foundation, Minneapolis, MN USA; 30 National Heart Centre Singapore, 5 Hospital Dr, Singapore, 169609 Singapore; 31 Department of Cardiology, Duke-NUS Medical School, Singapore, Singapore; 32 Department of Cardiology, Sengkang General Hospital, Singapore, Singapore; 33 Department of Emergency Medicine, Christchurch Hospital, Christchurch, New Zealand; 34 Department of Medicine, University of Otago, Christchurch, New Zealand; 35 Department of Emergency Medicine, University of Kansas Medical Center, The University of Kansas Health System, Kansas City, KS USA; 36 British Heart Foundation Centre for Cardiovascular Science, University of Edinburgh, Edinburgh, UK; 37 Department of Emergency Medicine, Baylor College of Medicine, Houston, TX USA

**Publisher correction: Clin Res Cardiol**

**https://doi.org/10.1007/s00392-025-02842-x**

When this article was published, the list of contributors was presented in the Electronic Supplementary Material, but not given in the XML version. This has now been corrected.

The Original Article has been updated.

