# Supplementary material for: Clinical performance of the next generation Elecsys Troponin T high-sensitivity Gen 6 assay in acute coronary syndrome (PERFORM-TSIX): study design
Source: Clin Res Cardiol. 2026 Jan 19;115(10):1807–18. doi: 10.1007/s00392-025-02842-x (PMC13562094; doi:10.1007/s00392-025-02842-x)
Supplement: Supplementary file 1 [file 392_2025_2842_MOESM1_ESM.docx]

# Clinical performance of the next generation Elecsys Troponin T high-sensitivity Gen 6 assay in acute coronary syndrome (PERFORM-TSIX): study design

## Clin Res Cardiol

Lori B. Daniels, Evangelos Giannitsis, Christian Mueller, Steven J.R. Meex, David Buehlmann, Dunja Kurtoic, Garnet Bendig, Mette Cole, Richard Body, Robert H. Christenson, Christa Cobbaert, Christopher R. deFilippi, Kai M. Eggers, Kenji Inoue, Allan S. Jaffe, Cian P McCarthy, James McCord, Johannes Tobias Neumann, Torbjørn Omland, Cynthia Papendick, Yader Sandoval, Jack Wei Chieh Tan, Martin P. Than, Raphael Twerenbold, Nicholas L. Mills, W. Frank Peacock

## Correspondence

Lori B. Daniels, MD, MAS, FACC, FAHA

Division of Cardiovascular Medicine, Department of Medicine, University of California, San Diego, La Jolla, California, United States

Email: [lbdaniels@health.ucsd.edu](mailto:lbdaniels@health.ucsd.edu)

# Supplementary Material

## Suppl. Table 1: Study sites

| Region | Institution name | Principal investigator | Institution address |
| --- | --- | --- | --- |
| US | El Centro Regional Medical Center | Christian Tomaszewski | 1415 Ross Ave El Centro, CA 92243, US |
|  | Atrium Health | Michael Runyon | Atrium Health Carolinas Medical Center- Main, 1000 Blythe Blvd, Dept. of Emergency Medicine, 306 Medical Education Building, Charlotte, NC 28203, US |
|  | Long Island Jewish Medical Center | Jonathan Gong | Emergency Department, 270-05 76th Ave New Hyde Park, NY 11040, US |
|  | Tampa General Hospital | David Wein | 1 Tampa General Circle, Tampa, FL 33606, US |
|  | Baylor College of Medicine | Sara Andrabi | 6620 Main St, Houston, TX, 77030-2348, US |
|  | Inova Fairfax Hospital | David Kopin | 3300 Gallows Road, Falls Church, VA, 22042, US |
|  | Aspirus Medical Group (affiliated with Aspirus Wausau Hospital) | Germain Larrain | 500 Wind Ridge Dr, Wausau, WI, 54401-4173, US |
|  | Oklahoma Heart Hospital | Naheem Tahirkheli | 5224 E I-240 Service Rd, Oklahoma City, OK, 73135-2607, US |
|  | University of Buffalo Erie County Medical center | Brian Clemency | 462 Grider St, Buffalo NY 14215, US |
|  | Dallas VA Medical Center | Shuaib Abdullah | 4500 South Lancaster Road, Dallas, TX, 75216, US |
|  | MultiCare Institute for Research & Innovation | Ravi Hira | 315 MLK Jr. Way, Tacoma, WA 98405, US |
|  | Henry Ford Health | Howard Klausner | 2799 W. Grand Blvd., Detroit, MI. 48202, US |
|  | Wake Forest Health Sciences | Simon Mahler | Medical Center Dr, Winston-Salem, NC 27157, US |
|  | Eastside Research Associates | Chad Crystal | 8301 161^st^ Ave NE, Redmond, WA 98052, US |
|  | Medical University of South Carolina | Gary Headden | 171 Ashley Ave, Charleston, SC 29425, US |
|  | Atrium Health Wake Forest Baptist, High Point Medical Center | Simon Mahler | 601 Elm St. High Point, NC 27262, US |
|  | Medical University Of South Carolina, MUSC Health Florence Medical Center | Gary Headden | 805 Pamplico Hwy Florence, SC 29505, US |
|  | Parkview Research | Peter Chaille | 10622 Parkview Plaza Dr. Fort Wayne, IN 46845, US |
|  | University of Kansas Medical Center | Chad Cannon | 3901 Rainbow Blvd. Mail stop 1019 Kansas City, US |
|  | Duke University | Alexander Limkakeng | 2301 Erwin Road, Durham, NC 27705, US |
|  | UCSD La Jolla ED | Vaishal Tolia | 9300 Campus Point Dr., La Jolla, CA 92037, US |
|  | Sentara Norfolk General Hospital | Barry Knapp | 600 Gresham Drive, Norfolk, VA 23507, US |
|  | University of Florida | Mohammad Massoomi | 1329 SW 16^th^ Street, PO Box 100186, Gainesville, FL 32610, US |
|  | Minneapolis Heart Institute Foundation | Yader Sandoval | 800 E 28th St, Minneapolis, MN 55407, US |
|  | Duke University - Satellite | Alexander Limkakeng  Emily Ko | 2301 Erwin Road, Durham, NC 27705, US |
|  | University at Buffalo / Buffalo General Hospital | Brian Clemency | 100 High St. Buffalo, NY, 14203, US |
| Europe (Austria) | Medical University Vienna | Jan Niederdöckl Filippo Cacioppo | Emergency Department 6 D, Medical University Vienna, Waehringer Guertel 18-20 1090 Vienna, Austria |
| Europe (Bulgaria) | MHAT "Heart and Brain" | Dobri Hazarbasanov | 1, "Zdrave' Str., Burgas 8000, Bulgaria |
|  | MHAT "Sartze I mozak", MHAT "Heart and Brain" | lana Simova | 2 Pier Kiuri Str. Pleven 5800, Bulgaria |
| Europe (Germany) | Charité-Universitaetsmedizin Berlin - Campus Charité Mitte (CCM) | Martin Moeckel | Ärztlicher Leiter Notfall- und Akutmedizin, Zentrale Notaufnahmen und Chest Pain Units Campus Charité Mitte und Campus Virchow-Klinikum, Charité - Universitätsmedizin Berlin Zentrale Notaufnahmen und Chest pain Units CVK/CCM, Augustenburger Platz 1, 13353 Berlin, Germany |
|  | Universitätsklinikum Hamburg-Eppendorf | Raphael Twerenbold | Martinistraße 52, 20246 Hamburg, Germany |
|  | Universitätsklinikum Heidelberg | Matthias Müller-Hennessen  Moritz Biener | Universitätsklinikum Heidelberg, Medizinische Klinik III, Im Neuenheimer Feld 410, 69120 Heidelberg, Germany |
| Europe (the Netherlands) | Catharina Ziekenhuis | Volkher Scharnhorst | Michelangelolaan 2, The Netherlands |
| Europe (Norway) | Akershus University Hospital (AHUS) | Torbjøm Omland | Akershus universitetssykehus HF, Sykehusveien 25, 1478 Nordbyhagen, , Norway |
| Europe (Spain) | Hospital Clinic of Barcelona | Oscar Mirö | C. de Villarroel, 170 Spain |
|  | Hospital Ålvaro Cunqueiro | Victor Jiménez Diaz | Hospital Álvaro Cunqueiro, Estrada De Clara Campoamor 341 Vigo, PO 36213, Spain |
| Europe (Switzerland) | University Hospital Basel | Christian Mueller | Universitätsspital Basel, Cardiovascular Research Institute of Basel (CRIB), Spitalstrasse 2, CH- 4056 Basel, Switzerland |
|  | Luzerner Kantonsspital | Michael Christ | Luceme Cantonal Hospital Spitalstrasse 16, Luzem 6000 Switzerland |
| Europe (UK) | NHS Lothian | Stephen Lynch | 49 Little France Crescent, EH16 4SU, Edinburgh, UK |
|  | Manchester University NHS Foundation Trust | Rick Body | Manchester University NHS Foundation Trust, Cobbett House, Manchester University NHS Foundation Trust, Oxford Road, Manchester, M13 9WL, UK |
| Japan | Omihachiman Cornmunity Med Center | Takuo Nakagami | 1379 Tsuchida-cho, Omihachiman-Shi, Japan |
|  | Tokyo Bay Urayasu, Ichikawa Medical Center | Tatsuya Nakama | 3-4-32 Todaijima, 2790001, Urayasu-shi, Chiba, Japan |
|  | Kurashiki Central Hospital | Kazushige Kadota | 1-1-1 Miwa, Kurashiki-shi, Okayama, 710-8602, Japan |
| China | Peking University First Hospital | Yong Huo  Yan Zhang | No.8 Xishiku Road, Xicheng District, Beijing, China |
|  | Zhongnan Hospital of Wuhan University | Yan Zhao | No. 169, Donghu Road, Wuchang District, Wuhan, Hubei Province, China |
|  | Dongguan People's Hospital | Yanhua Yang | No.3 Xinguchongwan Road, Wanjiang District, Dongguan, Guangdong Province, China |
|  | Tongren Hospital Shanghai Jiao Tong University School of Medicine-Shanghai Tongren Hospital | Zhaohui Qiu | 1111 Xianxia Road, Changning District, Shanghai, China |
|  | Tongji Hospital, Tongji Medical College of Huazhong University of Science and Technology | Li Yan | No. 1095, Jiefang Road, Qiaokou District, Wuhan City, Hubei Province, China |
|  | Sun Yat-sen Memorial Hospital, Sun Yat-Sen University | Longyuan Jiang | 33 Yingfeng Road,Haizhu Dst, Guangzhou, China |
|  | TEDA International Cardiovascular Hospital | Weiqiang Chen | No. 61, Third Avenue, Binhai new District, Tianjin City, 300457, China |

## Suppl. Table 2: Testing sites

| Region | Institution name | Principal investigator | Institution address |
| --- | --- | --- | --- |
| US | TRICORE REF LAB | Veronica Luzzi | 1001 Woodward Place, Albuquerque, NM 87102, US |
|  | University of Maryland | Robert Christenson | University of Maryland, College Park, MD 20742, US |
|  | UC Davis Medical Center | Nam Tran | 4301 X St, Sacramento, CA 95817, US |
|  | Washington University | Jennifer Powers Carson | 1 Brookings Dr, St. Louis, MO 63130, US |
| Europe (Germany) | MVZ Labor Dr. Limbach & Kollegen GbR | Peter Findeisen | MVZ Heidelberg, Im Breitspiel 16, 69126 Heidelberg, Germany |
| China | Peking University First Hospital | Yong Huo  Yan Zhang | No.8 Xishiku Road, Xicheng District, Beijing, China |
|  | Zhongnan Hospital of Wuhan University | Yan Zhao | No. 169, Donghu Road, Wuchang District, Wuhan, Hubei Province, China |
|  | Dongguan People's Hospital | Suxia Guo | No.3 Xinguchongwan Road, Wanjiang District, Dongguan, Guangdong Province, China |
|  | Tongren Hospital Shanghai Jiao Tong University School of Medicine-Shanghai Tongren Hospital | Huiming Sheng | 1111 Xianxia Road, Changning District, Shanghai, China |
|  | Tongji Hospital, Tongji Medical College of Huazhong University of Science and Technology | Liming Cheng | No. 1095, Jiefang Road, Qiaokou District, Wuhan City, Hubei Province, China |
|  | Sun Yat-sen Memorial Hospital, Sun Yat-Sen University | Zhaohui Duan | 33 Yingfeng Road,Haizhu Dst, Guangzhou, China |
|  | TEDA International Cardiovascular Hospital | Kegang Jia | No. 61, Third Avenue, Binhai new District, Tianjin City, 300457, China |

## Suppl. Table 3: Data collection

| **IN HOSPITAL** | |
| --- | --- |
| Demographics | - Sex (female, male) – assigned at birth and whether preferred gender is different from gender assigned at birth - Age - Ethnicity - Race |
| Medical history | - MI - CAD - Coronary intervention (PTCA/PCI) - CABG - Family history of MI/CAD (defined as 1^st^ degree relative, male age <55, female age <65) - Angina pectoris - Valvular heart disease - Prosthetic heart disease (heart valve replacement) - Congestive heart failure - Permanent pacemaker/ICD - Atrial fibrillation/flutter - Hypertension - Hyperlipidemia/dyslipidemia - Cerebrovascular disease - Peripheral arterial disease - Diabetes (If yes, diabetes type?) - Chronic kidney disease (If yes, on dialysis?) - Pulmonary disease (If yes, requiring prescription medication?) - Malignancy within the past 5 years (If yes, please specify) - Muscle/skeletal disease or injury currently under the care of a physician - Current pregnancy - Current illicit drug use - Smoking (current, former, never) |
| Medication (received within 7 days of presentation to the ED and/or during active study phase) | - ACE inhibitors - Angiotensin receptor blockers - Beta-blockers - Calcium channel blockers - Other antihypertensive drugs - Antiplatelet drugs (if yes, which of the following: aspirin, cilostazol, clopidogrel (Plavix), dipyridamole (Persantine), prasugrel (Effient), ticagrelor (Brilinta), other) - Anticoagulants - Thrombolytics/fibrinolytics (only in-hospital) - Diuretics - Neprilysin inhibitors - Antiarrhythmic drugs - Inotropes/vasopressors (only in-hospital) - Nitrates - Lipid-lowering drugs - Antidiabetic drugs - Supplements (received within 7 days of presentation to the ED and/or during active study phase), e.g., biotin |
| ED presentation | - Date and time of presentation to the ED - Date and time of symptom onset (first episode) - Date and time of symptom peak - Date and time of last episode - HEART score - Description of symptoms leading to an ED visit^a^ |
| Vital signs | - Height - Weight - Blood pressure - Heart rate - Oxygen saturation |
| ECG, chest imaging and additional diagnostic or therapeutic procedures (during the active study phase [study inclusion until discharge from the hospital] and until the 180-day follow-up period) | - Select ECGs - Chest X-ray - Chest CT - Additional therapeutic procedures during index visit - Echocardiogram - Cardiac CT - Cardiac MRI - Stress test - Invasive angiography   - PTCA   - CABG |
| Local cardiac marker and additional testing | - All cTn or other cardiac marker test results done in routine with date and time - Type of cTn or other cardiac marker assay used in routine and its corresponding cut-off value - Date and time of routine lab tests - Results with the following parameters (if measured in routine)   - Hemoglobin   - BNP/NT-proBNP   - Creatinine   - eGFR   - Bilirubin (China only)   - Triglycerides (China only) |
| ED discharge | - Date and time of ED discharge, i.e., the time the physician decides to discharge the patient, not the time the patient physically leaves the ED - ED disposition   - Discharged home   - Discharged to skilled nursing facility or rehabilitation   - Discharged to outpatient care/follow up   - Admitted to hospital   - Transfer to other hospital   - Left against medical advice   - Deceased - Diagnosis at ED discharge   - MI (STEMI, NSTEMI [type 1–4])   - Unstable angina   - Cardiovascular non-coronary artery disease   - Non-cardiac disease or symptoms of unknown origin |
| Hospital discharge | - Date and time of discharge from the hospital - Hospital discharge summary   - Home (self-care)   - Home (with home health services)   - Discharged to skilled nursing facility or rehabilitation   - Discharged to outpatient care/follow up   - Transfer to other hospital   - Left against medical advice   - Deceased - Diagnosis at hospital discharge   - MI (STEMI, NSTEMI [type 1–4])   - Unstable angina   - Cardiovascular non-coronary artery disease   - Non-cardiac disease or symptoms of unknown origin |
| In-hospital events (defined as events that happen after the index event during hospitalization, here defined as between ED discharge and hospital discharge)^b^ | - MACE   - MI (any MI type 1–5 after the index event)   - Cardiac death according to the definitions for cardiovascular and stroke outcomes ^[1]^   - Non-cardiac death - All-cause death |
| Blood collection and sample processing | - Date and time of every blood collection for the study (T0, T1, T2, T3, T6) - Recording of underfilling - Date and time of centrifugation - Number of aliquots - Date and time of freezing |
| Adverse events | - Start and end date and time - Severity (mild, moderate, severe, serious) - Relation to the blood draw - Action taken - Treatment - Outcome - Discontinuation from the study - Reporting responsibilities |
| **FOLLOW-UP (30 and 180 days)** | |
| Follow-up events (30 and 180 days) | - Hospitalization - MACE since index visit:   - MI (incl. type of MI for the 30-day follow-up)^b^   - Cardiac death according to the uniform definitions for cardiovascular and stroke outcomes ^[1]^   - Non-cardiac death - All-cause death |
| Follow-up events (30 days only) | - Diagnostic or therapeutic procedures performed (30-day follow-up only)   - ECG - Additional diagnostic or therapeutic procedures   - Echocardiogram, incl. date   - Cardiac CT, incl. date   - Cardiac MRI, incl. date   - Stress test, incl. date   - Invasive angiography, incl. date   - PTCA (urgent/routine), incl. date   - CABG (urgent/routine), incl. date |

^a^Description of symptoms leading to emergency department visit were listed as checkboxes and include: Chest Pain (including pressure, burning sensation across the precordium and epigastrium, pain that radiates to the neck, shoulder, upper abdomen, back, jaw); acute onset or worsening dyspnea; nausea – vomiting or indigestion; light-headedness or syncope; diaphoresis; generalized weakness or fatigue; none (provide details)/asymptomatic but ACS suspected; other/atypical symptoms leading to suspicion of ACS. If chest pain is checked, additional questions about the pain must be answered. ^b^In-hospital and follow-up events are site reported and are not adjudicated by the independent clinical events committee.

*ACE* angiotensin-converting enzyme, *ACS* acute coronary syndrome, *BNP* B-type natriuretic peptide, *CABG* coronary artery bypass graft, *CAD* coronary artery disease, *CT* computed tomography, *cTn* cardiac troponin, *ECG* electrocardiogram, *ED* emergency department, *eGFR* estimated glomerular filtration rate, *ICD* implantable cardioverter defibrillator, *MACE* major adverse cardiovascular events, *MI* myocardial infarction, *MRI* magnetic resonance imaging, *NSTEMI* non-ST segment elevation myocardial infarction, *NT-proBNP* N-terminal pro b-type natriuretic peptide, *PCI* percutaneous coronary intervention, *PTCA* percutaneous transluminal coronary angioplasty, *STEMI* ST segment elevation myocardial infarction

## Suppl. Table 4: TSIX investigators.

| **Region** | **Study** | **Principal Investigator** | **Institution name** |
| --- | --- | --- | --- |
| **US** | REF-TSIX | Michael B Jacobs | AMR Las Vegas |
|  |  | Gregg Lucksinger | Velocity Clinical Research – Cedar Park |
|  |  | Richard Powell | Velocity Clinical Research – Cincinnati |
|  |  | Daniel Johnson | Artemis Institute for Clinical Research - San Diego |
|  |  | Hugh Coleman | Covance CRU Daytona |
|  |  | Melanie Fein | High Point Clinical Trials Center |
|  |  | Joseph Surber | IACT Health |
|  |  | Jack Vu | Artemis Institute for Clinical Research – Riverside |
|  |  | Michael Hassman | Hassman Research Institute |
|  |  | John Hernandez | Sera Collection |
|  |  | Judith Kirstein | Velocity Clinical Research – Banning |
|  | PERFORM-TSIX | Christian Tomaszewski | El Centro Regional Medical Center |
|  |  | Michael Runyon | Atrium Health |
|  |  | Jonathan Gong | Long Island Jewish Medical Center |
|  |  | David Wein | Tampa General Hospital |
|  |  | Sara Andrabi | Baylor College of Medicine |
|  |  | David Kopin | Inova Fairfax Hospital |
|  |  | Germain Larrain | Aspirus Medical Group (affiliated with Aspirus Wausau Hospital) |
|  |  | Naheem Tahirkheli | Oklahoma Heart Hospital |
|  |  | Brian Clemency | University of Buffalo Erie County Medical center |
|  |  | Shuaib Abdullah | Dallas VA Medical Center |
|  |  | Ravi Hira | MultiCare Institute for Research & Innovation |
|  |  | Howard Klausner | Henry Ford Health |
|  |  | Simon Mahler | Wake Forest Health Sciences |
|  |  | Chad Crystal | Eastside Research Associates |
|  |  | Gary Headden | Medical University of South Carolina |
|  |  | Simon Mahler | Atrium Health Wake Forest Baptist, High Point Medical Center |
|  |  | Gary Headden | Medical University Of South Carolina, MUSC Health Florence Medical Center |
|  |  | Peter Chaille | Parkview Research |
|  |  | Chad Cannon | University of Kansas Medical Center |
|  |  | Alexander Limkakeng | Duke University |
|  |  | Vaishal Tolia | UCSD La Jolla ED |
|  |  | Barry Knapp | Sentara Norfolk General Hospital |
|  |  | Mohammad Massoomi | University of Florida |
|  |  | Yader Sandoval | Minneapolis Heart Institute Foundation |
|  |  | Alexander Limkakeng  Emily Ko | Duke University - Satellite |
|  |  | Brian Clemency | University at Buffalo / Buffalo General Hospital |
|  | Testing site | Veronica Luzzi | TRICORE REF LAB |
|  |  | Robert Christenson | University of Maryland |
|  |  | Nam Tran | UC Davis Medical Center |
|  |  | Jennifer Powers Carson | Washington University |
| **Europe (Austria)** | PERFORM-TSIX | Jan Niederdöckl  Filippo Cacioppo | Medical University Vienna |
| **Europe (Bulgaria)** | REF-TSIX | Emil Kolev | DCC Convex Ltd. |
|  |  | Todor Popov | Medical Center Excelsior |
|  |  | Andrey Petrov | MHAT Lyulin |
|  | PERFORM-TSIX | Dobri Hazarbasanov | MHAT "Heart and Brain" |
|  |  | lana Simova | MHAT "Sartze I mozak", MHAT "Heart and Brain" |
| **Europe (Germany)** | REF-TSIX | Luci Suganthi Magimaiseelan | Emovis GmbH -> new Futuremeds |
|  |  | Christine Kosch | Hausärztlich-diabetologische Praxis Christine Kosch |
|  |  | Gernot Klein | Apex GmbH |
|  |  | Kai Mehltretter | SIBAmed GmbH & Co KG |
|  |  | Joachim Sauter | Zentrum für klinische Forschung Allgäu Oberschwaben |
|  |  | Sybille Baumann | CRS Clinical Research Services Berlin GmbH |
|  | PERFORM-TSIX | Martin Moeckel | Charité-Universitaetsmedizin Berlin - Campus Charité Mitte (CCM) |
|  |  | Raphael Twerenbold | Universitätsklinikum Hamburg-Eppendorf |
|  |  | Matthias Müller-Hennessen  Moritz Biener | Universitätsklinikum Heidelberg |
|  | Testing site | Peter Findeisen | MVZ Labor Dr. Limbach & Kollegen GbR |
| **Europe (the Netherlands)** | PERFORM-TSIX | Volkher Scharnhorst | Catharina Ziekenhuis |
| **Europe (Norway)** | PERFORM-TSIX | Torbjøm Omland | Akershus University Hospital (AHUS) |
| **Europe (Poland)** | REF-TSIX | Urszula Wilczek-Kosowska | Krakowskie Centrum Medyczne FutureMeds |
|  |  | Ewa Zimmer- Satora | Małopolskie Centrum Kliniczne |
|  |  | Ewa Krecipro-Nizińska | FutureMeds sp. z o.o. |
| **Europe (Spain)** | PERFORM-TSIX | Oscar Mirö | Hospital Clinic of Barcelona |
|  |  | Victor Jiménez Diaz | Hospital Ålvaro Cunqueiro |
| **Europe (Switzerland)** | PERFORM-TSIX | Christian Mueller | University Hospital Basel |
|  |  | Michael Christ | Luzerner Kantonsspital |
| **Europe (UK)** | PERFORM-TSIX | Stephen Lynch | NHS Lothian |
|  |  | Rick Body | Manchester University NHS Foundation Trust |
| **Japan** | REF-TSIX | Shunji Matsuki | Fukouka Mirai Hospital |
|  |  | Takashi Eto | Hakata Clinic |
|  |  | Takanori Tanaka | Nishikumamoto Hospital |
|  |  | Ryuzo Hanada | Sumida Hosptial |
|  | PERFORM-TSIX | Takuo Nakagami | Omihachiman Cornmunity Med Center |
|  |  | Tatsuya Nakama | Tokyo Bay Urayasu, Ichikawa Medical Center |
|  |  | Kazushige Kadota | Kurashiki Central Hospital |
| **China** | REF-TSIX and testing site | Shan Jing | Beijing Anzhen Hospital, Capital Medical University |
|  |  | Shuwen Yu | Qilu Hospital of Shandong University |
|  |  | Hongxuan Zhang | Guangdong Provincial People's Hospital |
|  |  | Jun Zhang | SIR RUN RUN SHAW HOSPITAL, Zhejing University School of Medicine |
|  | PERFORM-TSIX and testing site | Yong Huo  Yan Zhang | Peking University First Hospital |
|  |  | Li Yan | Tongji Hospital, Tongji Medical College of Huazhong University of Science and Technology |
|  |  | Longyuan Jiang | Sun Yat-sen Memorial Hospital, Sun Yat-Sen University |
|  |  | Weiqiang Chen | TEDA International Cardiovascular Hospital |
|  | REF-TSIX, PERFORM-TSIX and testing site | Jianying Huang  Yan Zhao | Zhongnan Hospital of Wuhan University |
|  |  | Ling Guan  Yanhua Yang | Dongguan People’s Hospital |
|  |  | Qin Zhang  Zhaohui Qiu | Shanghai Tongren Hospital |

# References

1.	Hicks KA, Mahaffey KW, Mehran R, Nissen SE, Wiviott SD, Dunn B, et al. 2017 cardiovascular and stroke endpoint definitions for clinical trials. Journal of the American College of Cardiology. 2018;71(9):1021–34.
